# An Integrated Approach for the Monitoring of Brain and Autonomic Response of Children with Autism Spectrum Disorders during Treatment by Wearable Technologies

**DOI:** 10.3389/fnins.2016.00276

**Published:** 2016-06-21

**Authors:** Lucia Billeci, Alessandro Tonacci, Gennaro Tartarisco, Antonio Narzisi, Simone Di Palma, Daniele Corda, Giovanni Baldus, Federico Cruciani, Salvatore M. Anzalone, Sara Calderoni, Giovanni Pioggia, Filippo Muratori, Michelangelo Study Group

**Affiliations:** ^1^Institute of Clinical Physiology, National Research Council of ItalyPisa, Italy; ^2^Department of Clinical and Experimental Medicine, University of PisaPisa, Italy; ^3^Institute of Applied Sciences and Intelligent Systems “Eduardo Caianiello”, National Research Council of ItalyMessina, Italy; ^4^Department of Developmental Neuroscience, IRCSS Stella Maris FoundationPisa, Italy; ^5^Department of Information Engineering, University of PisaPisa, Italy; ^6^HelpyLife TechnologyOristano, Italy; ^7^I+ srlFlorence, Italy; ^8^Institute of Intelligent Systems and Robotics, University Pierre and Marie CurieParis, France

**Keywords:** Autism Spectrum Disorders (ASD), quantitative EEG (QEEG), electrocardiogram (ECG), wearable sensors, monitoring, naturalistic, personalization, imitation

## Abstract

Autism Spectrum Disorders (ASD) are associated with physiological abnormalities, which are likely to contribute to the core symptoms of the condition. Wearable technologies can provide data in a semi-naturalistic setting, overcoming the limitations given by the constrained situations in which physiological signals are usually acquired. In this study an integrated system based on wearable technologies for the acquisition and analysis of neurophysiological and autonomic parameters during treatment is proposed and an application on five children with ASD is presented. Signals were acquired during a therapeutic session based on an imitation protocol in ASD children. Data were analyzed with the aim of extracting quantitative EEG (QEEG) features from EEG signals as well as heart rate and heart rate variability (HRV) from ECG. The system allowed evidencing changes in neurophysiological and autonomic response from the state of disengagement to the state of engagement of the children, evidencing a cognitive involvement in the children in the tasks proposed. The high grade of acceptability of the monitoring platform is promising for further development and implementation of the tool. In particular if the results of this feasibility study would be confirmed in a larger sample of subjects, the system proposed could be adopted in more naturalistic paradigms that allow real world stimuli to be incorporated into EEG/psychophysiological studies for the monitoring of the effect of the treatment and for the implementation of more individualized therapeutic programs.

## Introduction

Recent advances in neuroimaging and other less-invasive neurophysiological monitoring systems allow researchers to explore the relationship between neurophysiological signals, neurodevelopmental disorders and behavioral changes. In particular, atypical patterns of brain activity and connectivity have been documented in children with Autism Spectrum Disorders (ASD) and are the basis of impaired and atypical behaviors (Belmonte et al., 2004; Geschwind and Levitt, 2007; Coben et al., 2008; Cantor and Chabot, 2009).

Electroencephalography (EEG) is a non-invasive technique able to identify dysfunction in various brain regions in autistic individuals. EEG measurements can be investigated in the frequency domain, and it has been convincingly demonstrated that assessing specific frequencies can yield insights into the functional correlations between brain regions. The EEG patterns analysis in the frequency domain is known as Quantitative EEG (QEEG). Commonly QEEG has been used to capture electrical patterns at the scalp surface, which primarily reflect cortical electrical activity or “brainwaves” (Tong and Thakor, 2009). Recently, several studies have used QEEG as a tool for neurophysiological assessment of children with ASD during resting state condition or specific tasks (for a review see Billeci et al., 2013). Interestingly, QEEG measurements can provide a mean to establish treatment efficacy (Dawson et al., 2012). According to the above-mentioned evidence, QEEG provides sufficient sensitivity and specificity to be worthy of consideration for use in the diagnosis, treatment and outcome evaluation of neurodevelopmental disorders.

ASD are also characterized by altered autonomic function, which plays an important role in the regulation of behaviors during social interaction. Physiological parameters and vital signs including heart rate, systolic and diastolic pressure, pulse rate, skin conductance, body temperature, and fingertip temperature can be used as cues of autonomic functions. In particular, heart rate variability (HRV) is a well-recognized method to assess the cardiac autonomic balance of the autonomic nervous system (Goldberger, 1999; Friedman, 2007). Although a few parameters, such as the HRV, are influenced by physical activity, nevertheless they have been increasingly used to measure the activity of the autonomic nervous system and to study neurophysiological responses in naturalistic settings (Goodwin et al., 2006). Other studies have shown an extremely high autonomic arousal in individuals with ASD even though they appeared to be outwardly calm (Hirstein et al., 2001; Hoch et al., 2010; Ming et al., 2011).

One of the limitations of the physiological parameters' assessment protocol is the artificial setting in which signals are usually acquired, which may or may not be closely related with naturally occurring stimuli. Therefore, these protocols induce both systematic and non-systematic biases to the experimental outcome eluding the actual nature of the data. Furthermore, they do not allow an ecological assessment and monitoring during treatment or even at home.

The recording of physiological signals [such as electroecephalogram (EEG) or electrocardioram (ECG)] during treatment in a semi-naturalistic setting may reveal important cues about the engagement, the arousal and emotional state and can support clinicians during therapeutic sessions in the implementation of the most appropriate personalized treatment plan.

The study of engagement is particularly relevant in ASD as deficits in this field (that is when the child is not able to share his/her attention to the other) emerge very early, in the second half of the first year of life, leading to reduced engagement with social stimuli, and subsequently reduced opportunities for social learning (Dawson et al., 1998; Mundy and Neal, 2000; Chevallier et al., 2012). These early deficits may thus have cascading effects on social communication development in successive years. In order to contrast these cascading effects evidence-based models (i.e., Early Start Denver Model) suggest building therapeutic tasks around specific engagement skills (i.e., imitation or joint attention) as core of the intervention (Billeci et al., 2016; Bono et al., 2016). During these tasks in which there is a social interaction with a therapist, it is possible to explore the response to social engagement and emotion regulation in response to social disengagement (that is when the child is not involved in social attention). In particular in the last years some studies have been performed, which provide evidences that physiological signals could constitute an important marker of social functioning and well-being that differentiate between engagement and disengagement during social interactions or treatment sessions (Patriquin et al., 2013; Di Palma et al., 2016; Shahrestani et al., 2016).

It is important to underline that monitoring physiological parameters during therapeutic sessions or during daily basis routines is definitely more challenging than monitoring the same parameters under controlled conditions. It is mandatory for example that the equipment should not interfere with the activities performed by the children during the treatment session. Wearable systems and wireless technologies can overcome this problem allowing monitoring subjects in an unobtrusive way (Varshney, 2005, 2006; Yilmaz et al., 2010).

The present study aims at describing and discussing the implementation of a wearable technology system that can be used during naturalistic interactions between a child and a clinician during a treatment session for the monitoring of brain and autonomic response. A preliminary application in a sample of children with ASD is presented. This work has been carried out within the framework of MICHELANGELO, a project funded by the European Commission (FP7- ICT-288241) (http://www.michelangelo-project.eu/).

The combined analysis of QEEG and ECG signals in semi-naturalistic settings enables simultaneous examination of neurophysiological and physiological correlates while the child is engaged in socio-emotive interactions. This system is able to provide (i) ecologically synchronized quantitative measurements of brain signals, and (ii) autonomic responses not measurable with traditional research methods within a natural environment. For this purpose, wearable technologies for EEG and ECG have been used, which are suitable for young children with ASD. The system proposed will allow a reliable indication of brain activity and autonomic status in a naturalistic setting contributing toward the implementation of more individualized and effective treatment for children with ASD.

## Materials and methods

### Participants and paradigm

The system was tested with five children with ASD (all males, age range = 6–8 years, mean age = 7.2 ± 0.83 years) (Table 1). The study was approved by the IRCSS Stella Maris Foundation's Ethical Committee and all the parents signed a written consent form to participate.

**Table 1:** **Participants characteristics**.

**Subjects**	**Age in years**	**Diagnosis**	**ADOS-2 Module**	**ADOS (CS)**	**WISC-IV-FSIQ**	**VABS-II Composite**
Child 1	7	Autism Spectrum	2	6	117	75
Child 2	8	Autism Spectrum	3	6	98	76
Child 3	6	Autism Spectrum	3	7	97	85
Child 4	7	Autism Spectrum	3	5	84	76
Child 5	6	Autism Spectrum	3	6	123	85

*CS, Comparison Score; WISC-FSIQ, Wechsler Intelligence Scale for Children—Full Scale Intelligence Quotient; VABS, Vineland Adaptive Behavior Scale*.

The ASD diagnosis was formulated according to the DSM-5 criteria (APA, 2013) and confirmed by the Autism Diagnostic Observation Schedule-2 (ADOS-2, Lord et al., 2012) and the Autism Diagnostic Interview-Revised (ADI-R, Lord et al., 1994).

The ADOS-2 diagnostic algorithm also provides an algorithm for computing the comparison score (CS), a measure of the severity of autism-related symptoms. The CS ranges from 1 to 10, where 1 indicates minimal-to-no evidence of autism-related symptoms and 10 indicates a high level of impairment.

The adaptive behavior was assessed according to the Vineland Adaptive Behavior Scale-II (VABS-II, Sparrow et al., 2005). A multidisciplinary team—including a senior child psychiatrist, and two clinical child psychologists experienced in ASD—conducted the diagnostic assessment during a 5-day extensive evaluation. The Wechsler Intelligence Scales for Children-IV (WISC-IV) were used to assess the Full Scale Intelligence Quotient (FSIQ).

**Figure 1:**
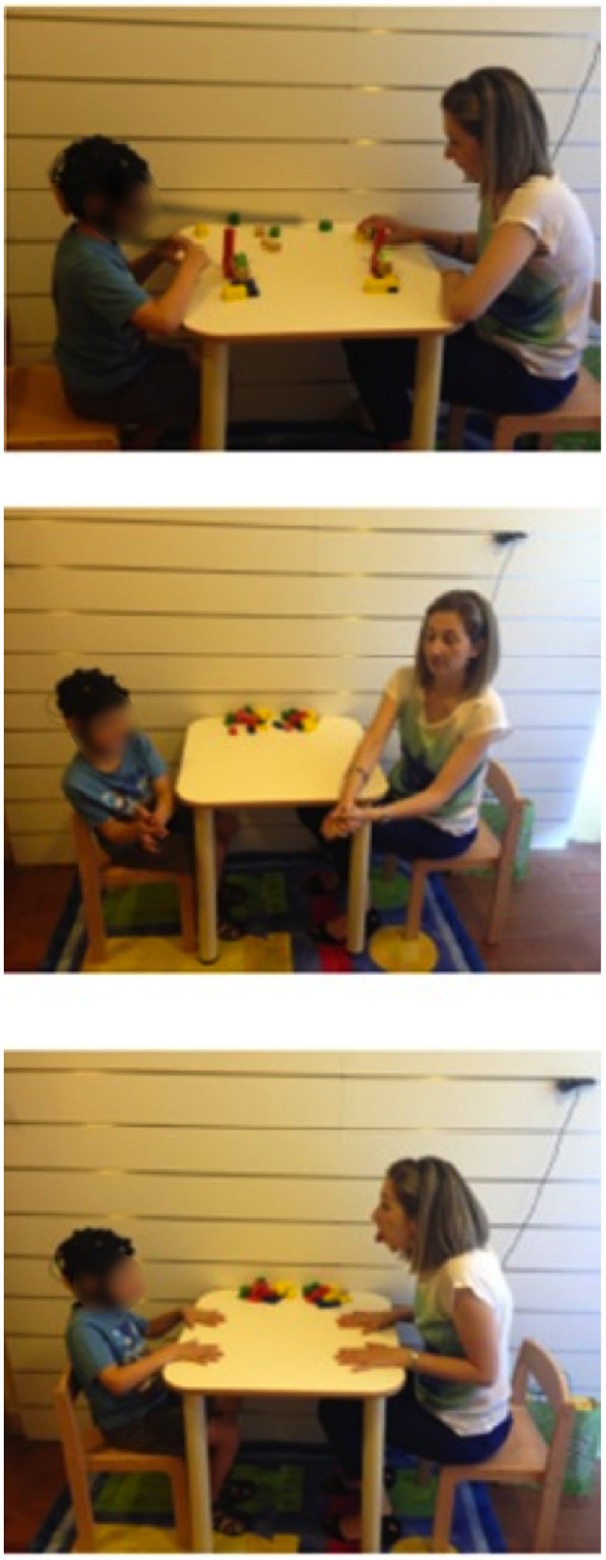
**Experimental paradigm**.

The children involved in the experiment were monitored during one therapy session in clinic with the integrated system. The semi-naturalistic experimental paradigm was based on imitation (IM) in the context of a play-based setting (Figure 1). Each session took about 15 min. During all the session the children remained seated on a chair playing at a table with the therapist in order to limit artifacts in the data acquired.

The IM phase is realized according to three different tasks in which the therapist asks to the child to imitate what she is doing. In task 1, the therapist uses blocks, play-doh, or sheets and markets in order to elicit functional imitation in the child; in task 2 the therapist suggests the child gestural and symbolic imitation; in task 3 the therapist elicits facial expressions in the child. Each task was repeated sever times in order to have several segments of the signals, which could be mediated to have more consistent results. During the experimental sessions data brain, autonomic and video data were recorded and analyzed in post-processing with the integrated platform described in the following paragraphs.

### Design

#### General overview

**Figure 2:**
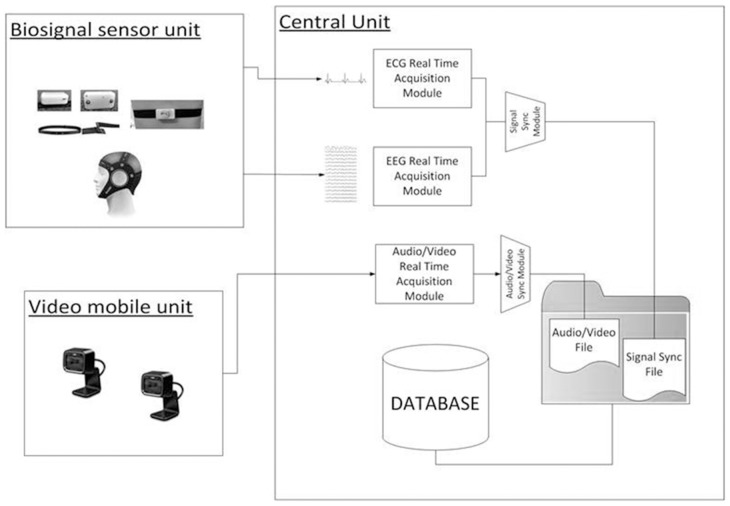
**Architecture overview**.

The overall architecture of the integrated system is shown in Figure 2. It mainly consists of three modules: the biosignal sensor unit, the video mobile unit, and a Central Unit (CU).

The main function of the biosignal sensor unit is the wearable and wireless acquisition of EEG and ECG signals while allowing the child to interact with the therapist. The unit is composed by the Enobio wireless device (STARLAB, Barcelona, Spain) for EEG recording (Cester et al., 2008) and by a wireless ECG chest belt (Solar et al., 2012).

The integrated system is also provided of data analysis toolboxes for the processing of the recorded video, EEG and ECG signals. The collected data can be loaded and queried for data visualization, segmentation, and feature extraction. The toolboxes are developed as a research tool in order to investigate physiological and behavioral parameters correlated with behaviors of the child during treatment sessions. The main components of the system are described in the following paragraphs (for details about EEG and ECG feature extraction see Supplementary Materials).

#### Biosignal sensor unit

Enobio offers a high degree of unobtrusiveness (easy to use, wearable, only 65 grams weight). Each active digital electrode with a transduction interface and electrode is able to acquire, digitize, and transmit the signal on site in order to reduce the environmental noise while recording data away from the lab or controlled environments.

The system continuously records EEG signals over 19 channels positioned according to the 10/10 standard scheme and two references (placed on the mastoid), at 500 Hz with a 16-bit accuracy. Enobio is equipped both with gel and dry electrodes. While gel electrodes provide a better contact with the skin and lower impedance, their positioning can be very long and uncomfortable for the child. In this setting dry electrodes are chosen as they offer a shorter and easier set-up time comparable with gel electrodes, which is particularly important with children with ASD. Several articles indeed show that dry electrodes can yield performance comparable to gel electrodes (Zander et al., 2011; Guger et al., 2012). Data from Enobio are recorded by a dedicated software (NIC, Neuroelectrics).

The chest belt for ECG acquisition is a wearable device designed by our group based on the Shimmer® (Burns et al., 2010) wireless base module, which is CE certified and validated prior to the present study in a group of healthy subjects (Solar et al., 2012).

The system is powered by a 3.6 V rechargeable battery, which allows up to 7 h of continuous monitoring per charge. The system sample frequency can be set from a minimum of 100 Hz up to a maximum of 500 Hz. The hardware module was tailored to be compliant with the common cardio-fitness Polar™ or Adidas™ chest straps in order to gain in ergonomics, to be lightweight and allow long-term monitoring. These straps are fully washable and integrate dry electrodes applied directly to the patient's skin for single-lead acquisitions.

#### Video mobile unit

The video mobile unit is made up by two environmental cameras with a frame rate of 25 fps and a resolution of 640 × 480 for video recording. The cameras are wired and synchronized with the system to contextualize the neurophysiological parameters with the behavior of the child. With the aim of building an exploitable research tool to investigate EEG signal, inexpensive webcam were used as video capture devices (Microsoft LifeCam HD-5000).

#### Central unit

**Figure 3:**
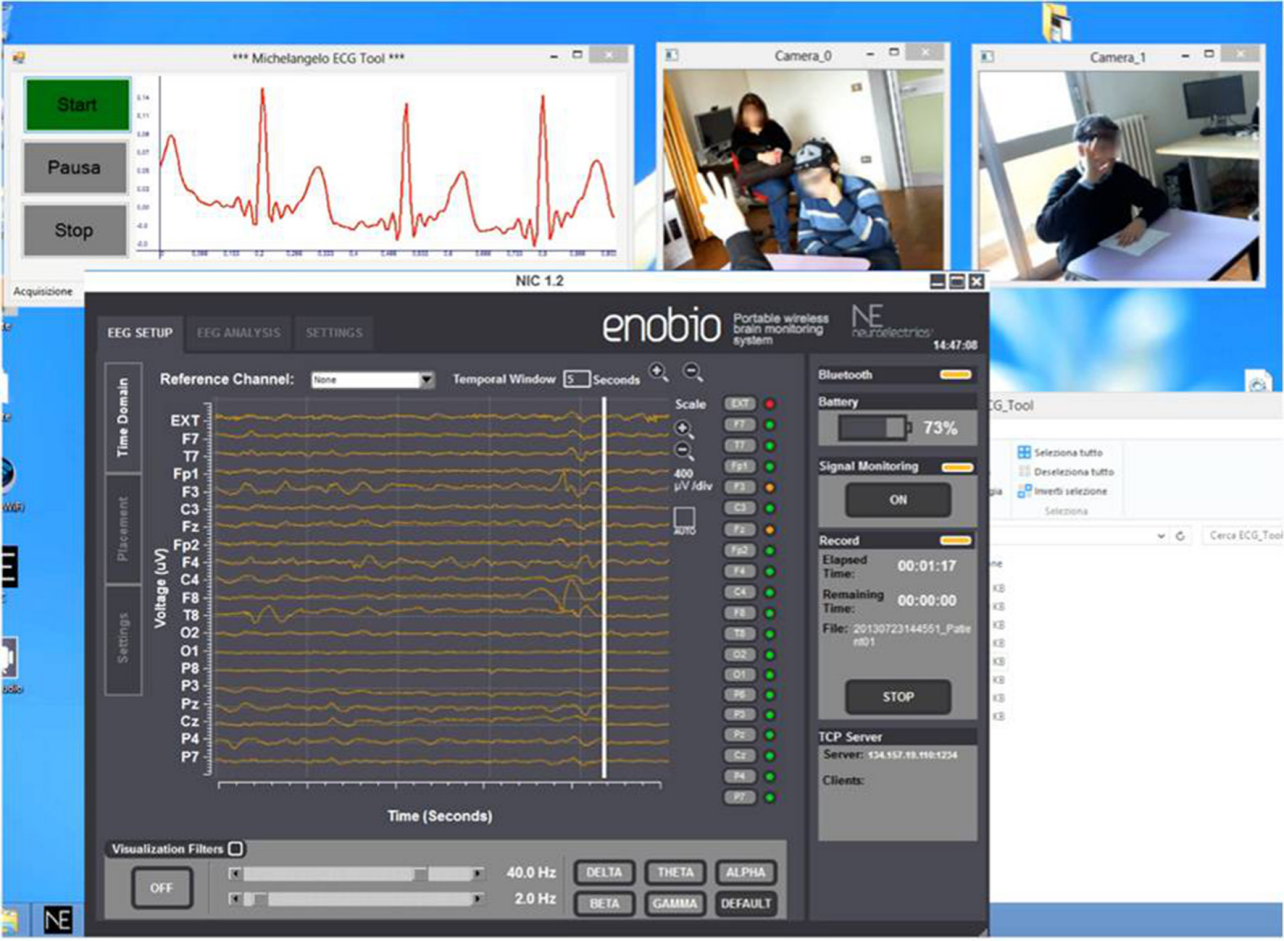
**Real-time acquisition of two camera video, ECG signal, and EEG signal during a therapeutic session**.

The CU (OS: Win8, RAM: 4 GB, CPU: Intel Core i5-3450—3.1 GHz—4 core) is positioned in the monitoring room and enables researchers to monitor the child during therapy and the neurophysiological signals. The monitoring room is located next to the therapy's room allowing the Bluetooth data transmission from the biosignal sensor unit and the video mobile data recording. Raw data are real-time displayed within *four* windows (ECG, EEG, Videos) as shown in Figure 3.

The CU manages the connection between the monitoring interface and the set of biosignal sensors and video mobile units, in particular the CU is responsible for notifying the “Start Session” message to all the recording units (RUs) involved and initializing each new session. All the communication between the CU and the RUs is transmitted in a validated XML format v1.0. The application manages security and privacy, real-time streaming and data synchronization. In particular the synchronization among the different devices is guaranteed by the fact their applications run in the same system and are launched simultaneously by an application running on the CU.

After data collection, the data analysis toolboxes running in the CU are able to analyze and process the collected data off-line by combining different data as a whole data source.

#### Video analysis toolbox

The video analysis toolbox allows the labeling of the children's behaviors in the recorded session and is based on the Dante annotation tool (Cruciani et al., 2011) customized for the labeling of behaviors of children with ASD.

Some of the behavioral features extracted are considered as instantaneous features (i.e., gesture), while others represent a state of the subject that persists in time (i.e., engagement).

**Figure 4:**
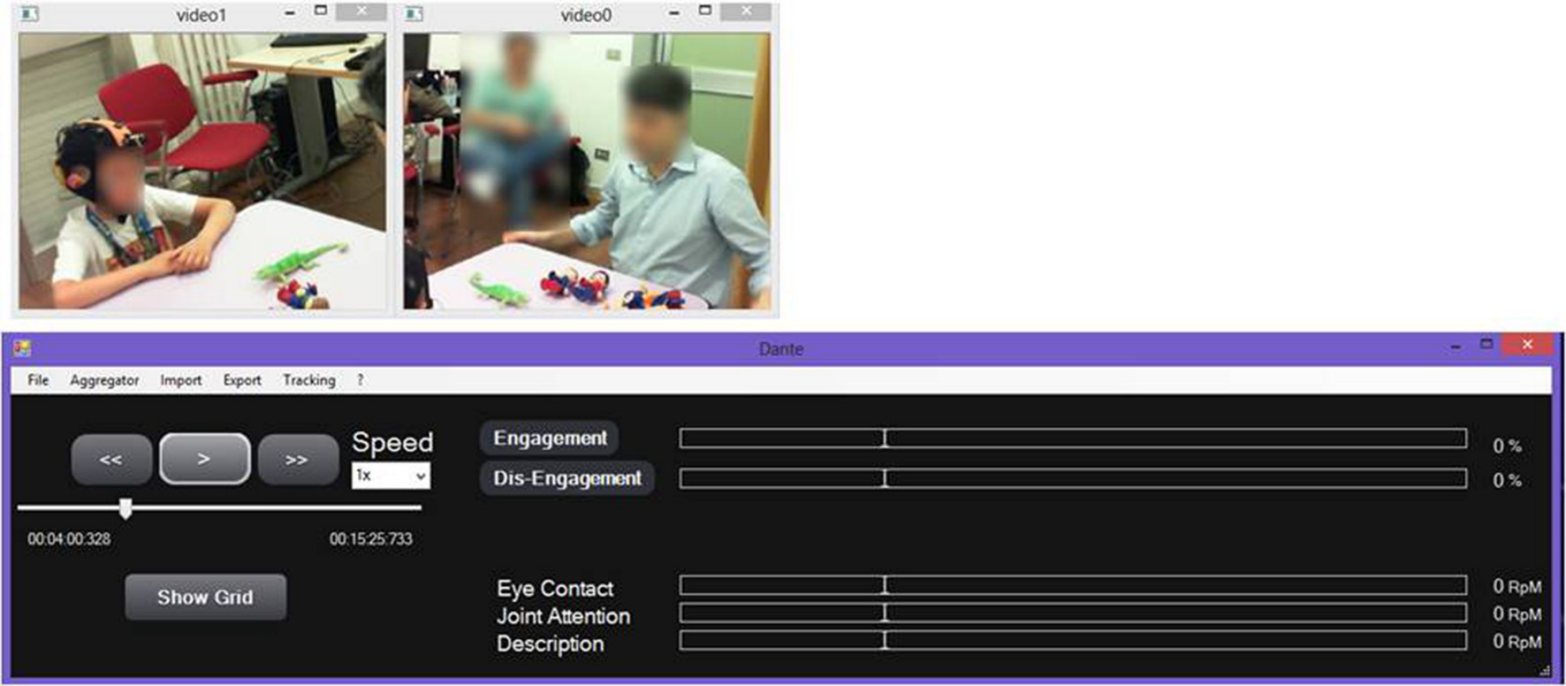
**Graphical User Interface for re-play and annotation of the recorded video session**.

The video analysis tool provides a Graphical User Interface (GUI) (Figure 4) to re-play the video of a recorded session, while examining annotated data and the integration of EEG/ECG data.

A manual annotation grid, referring to the child and the therapist, allows behavioral analysis of the therapeutic session, as defined in the coding scheme.

The grid distinguishes between “States” and “Behaviors.” States identify behavioral states of a subject that persist for a given duration in time, for example Engagement and Dis-Engagement. States are always considered as mutually exclusive. In contrast, Behaviors are always considered as instantaneous events, meaning that the corresponding event will have the same timestamp for Start Time and End Time.

The Video Analysis interface produces an “Event File,” an. xls file containing the information about the annotated events (type of state/behavior, Start Time, End Time). Through the annotation of these events, each therapy session can be analyzed to provide contextual information on significant behaviors of the child, useful for exploratory analysis of EEG/ECG signal.

#### EEG analysis toolbox

EEG signals are first-pre-processed using EEGLAB Toolbox (Delorme and Makeig, 2004) to remove noise and artifacts. The recorded EEG signals are high passed at 0.5 Hz to get rid of noise from breathing and low passed at a cut-off frequency of 70 Hz to get rid of the high frequency noise. A Notch filter (45–55 Hz) is also applied to remove power line interference.

Ocular and muscular artifacts are first removed by visual inspection. In particular blink artifacts are identified as segments of data having deflection >150 μV, while ocular flutters, or muscle movement artifacts as segments having deflections of 50 μV relative to baseline. Segments containing these artifacts are excluded from the following analyses. In addition bad channels are removed using the “channel statistics” tool of EEGLAB. On the basis of these statistics “bad” channels were considered as the ones that had distributions of potential values that were further away from a Gaussian distribution than other scalp channels, and they were remove from further analysis.

After artifacts removal baseline signal is removed form data acquired during the task.

After-pre-processing, EEG data are imported in a home-made Matlab analysis toolbox for QEEG analysis.

First, the Power Spectral Density (PSD) is evaluated by transforming the signal from the time domain to the frequency domain using the Welch method (Welch, 1967). Then the absolute and the relative power of each band (delta: 1–4 Hz, theta: 4–7 Hz, alpha: 8–13 Hz, beta: 14–29 Hz, and gamma: 30–70 Hz) are computed for each electrode. Relative powers are usually more reliable than absolute powers because they show less variability among different subjects and they are less affected by artifacts (Duffy, 1986). Inter-hemispheric asymmetry is computed by the Brain Symmetry Index (BSI) (van Putten et al., 2004). The BSI is calculated within each EEG band considering the total power in each region (frontal, temporal, central, parietal, and occipital) of the left and right hemisphere (sum of the electrodes).

The connectivity between brain regions is estimated with the calculation of coherence, which gives an estimation of the linear correlation between two signals collected at two different scalp points as a function of frequency (Otnes and Enochson, 1972). Coherence is calculated for each pair of electrodes in each frequency band.

#### ECG analysis toolbox

ECG signals are pre-processed through a stepwise filtering process aimed at removing typical ECG artifacts and interferences. In particular, the baseline wander due to body movements and respiration artifacts are removed using a cubic spline 3rd order interpolation between the fiducial isoelectric points of the ECG (Jane et al., 1992). The power line interference and muscular noise are removed using an IIR (infinite impulse response) notch filter at 50 Hz and an IIR low pass filter at 40 Hz. In the final step of the pre-processing phase the Pan-Tompkins method is applied to detect the QRS complexes (Pan and Tompkins, 1985). The tachogram and the HRV are extracted according to the International Guidelines of HRV (Task Force of the European Society of Cardiology the North American Society of Pacing Electrophysiology, 1996). The tachogram is a vector whose elements represent the beat-to-beat interval between *two* adjacent R peaks in the ECG allowing the definition of useful features for a further quantitative analysis. At this step the tachogram signal might not be suitable yet for a proper features extraction due to the presence of possible residual movement artifacts and outliers, which can be easily detected by visual inspection. Artifact are visually identified and removed. In order to prevent the signal from excessive shortening, the user should operate a careful artifacts selection choosing the interval to remove as tight as possible. Outliers are replaced by division or summation. Division is applied when the outlier is determined by a failure to detect an R-peak while summation while it is caused by faulty detections of *two* or more peaks within a period representing the R-R interval.

After pre-processing significant features, which could give an indication of the engagement of the child during the therapy are extracted from the ECG signal. In particular the Heart Rate (HR), the Root Mean Square of the Successive Differences (RMSSD), and the Respiratory Sinus Arrhythmia (RSA) are selected. The HR measures the number of poundings of the heart occurring in a specific lapse of time and it is typically expressed as beats per minute (bpm). The RMSSD, indicator of vagal activity, was chosen as a time domain measure of the HRV. The RSA refers to the periodic fluctuations in HR that are linked to breathing. RSA is largely determined by vagal influences on the heart, providing a noninvasive index of parasympathetic activity, social functioning, and cognitive performances (Patriquin et al., 2013, 2014). The RSA component from tachogram signal is extracted using the Empirical Model Decomposition (EMD) (Balocchi et al., 2004).

### Data collection and measurement definition

Data were collected using the technological platform described above during a therapeutic session of each participant included in this study. The Biosignal Sensor Unit and the Video Mobile Unit were connected to the CU and a new acquisition session was recorded for each child. The EEG data were acquired using Enobio at 500 Hz while for ECG signals a sampling frequency of 200 Hz was chosen to avoid too much noise and to optimize data transfer.

Data collected were analyzed in post-processing. Each recording session was first imported in the “Video analysis toolbox” and then coded by clinicians who reviewed the videos to define the events of interest. In particular, in this study we focused on the alternation of the “States,” i.e., the Engagement and the Disengagement, during the therapeutic session.

Two coders were trained to use the coding system, by observing and coding videos of children with ASD, who were not included in the study. The 1 month training period enabled the coders to become familiar with the meaning of the features, to identify them correctly, and to acquire ability in coding procedures. The coders were required to achieve a satisfactory level of agreement between them (Cohen's Kappa ≥0.70) and with two expert clinicians (AN and SC) for each Event behavior (imitation) and for each State behaviors (Engagement and Disengagement).

The tolerance window regarding time discrepancy between coding was set at 1 s for event behaviors and at 3 s for state behaviors. The coding recorded outside these windows was reported as a coding error and was considered a disagreement. Intercoder agreement was calculated for each item, and the values of *k* were ≥0.70.

The mean Intercoder reliability showed satisfactory agreement (*k* = 0.81). In order to verify the ongoing agreement, 25% of the collection of sequences were coded by both coders.

The mean Intercoder agreement calculated on these sequences was *k* = 0.84, and the values of *k* were ≥ 0.70. In all cases of discordance between coders, the expert coders advice was used.

For a definition of a baseline, both for EEG and ECG, signals were acquired for about 3 min before the beginning of the task.

The Event File containing the information about coding was used to extract EEG and ECG segments in order to link different physiological patterns with different states of the children.

For the EEG analysis, data acquired were divided in “Engagement” and “Disengagement” states using the information contained in the Event File. First the segments were imported in the EEGLAB Toolbox and pre-processed. The baseline signal was removed from the two state signals. The cleaned segments were then imported in the QEEG analysis GUI to extract features of interest. The proportion of data retained after cleaning was not different between Engagement and Disengagement phases (Engagement: 7.34%, Disengagement: 7.85%). Finally, features extracted for each segment type of each type of State were averaged.

For the purpose of data reduction, *four* EEG lead-groupings were considered to compute coherence (Coben et al., 2008; Machado et al., 2015; see Supplementary Materials).

ECG data were imported in the ECG analysis GUI and after pre-processing relevant features were extracted. The tool generates plots, which show the trend of the features over time, and also stores data for a further numerical analysis. To evaluate how the therapeutic stimuli influence the cardiac activity, physiological events were defined for each feature when the parameter undergoes or exceeds established values. A crucial step lies in the definition of thresholds, which denote the occurrence of a physiological event related to the feature of interest. Reference values were customized for each child at each acquisition since environmental, subjective, and current emotional states could heavily affect the HR baseline.

The values for “higher HR” and “lower HR” were respectively calculated by the 90th and the 5th percentile of the baseline signal. The event of “lower RMSSD” referred to values undergoing the 20 ms threshold and denoted more effective participation from the child (Althaus et al., 1999). The threshold for “lower RSA” ranged in an interval from 6.3 to 6.5 ln(ms^2^) (Porges et al., 2013).

For each State the mean and the standard deviation of the feature extracted as well as the percentage of physiological events detected were recorded. The percentage of physiological events was evaluated as the ratio of occurrences of the events to the total engagement time The approach described allowed to evaluate, both with a visual and a quantitative method, the physiological response of the child to the stimuli received with distinct reference to the different phases identified during the treatment.

### Data analysis

A preliminary statistical analysis was performed to evaluate differences in the features extracted between Engagement and Disengagement. Statistical analyses were performed in SPSS (SPSS Inc., Chicago, IL, USA). The Shapiro-Wilk test was applied to test the normality of the variables. For normal distributed variables a paired-samples *t*-test was applied, while for nonparametric variables a Wilcoxon-test was adopted. Results were considered significant for *p* < 0.05.

## Results

### Feasibility evaluation

The children did not show sensory-motor and/or behavioral issues in wearing the devices and completing all the tasks, administered within a friendly, and supportive environment without any difficulties or constraints.

We used labeled data generated through Manual Annotation to validate the system. In particular, the synchronization between Video and the other signals was crucial, since the video was the reference for the validation process. However, the required constraints on synchronization were not particularly strict considering that:

- the Video footage, which is the reference, has a frame rate considerably smaller compared to the EEG sample rate (25 vs. 500 Hz);
- even in behavioral annotation performed by a trained psychologist, a lag of 1 s is acceptable.

Therefore, the residual synchronization error due to the latency time between the data sampling and the reception by the synchronized machine is marginal considering that, whatever is the instant annotated as the start of the State, we considered the whole duration of the State for the analysis.

The synchronization tests have been performed by inserting a spike in the signal and comparing the video timestamp with the sensor data timestamps. The resultant accuracy with this setup is in the order of tenths of second, which is acceptable for our purpose.

### EEG results

Although each child showed his own individual pattern of brain activity and connectivity, all the children displayed visible changes of EEG pattern between the Engagement and Disengagement phases. Some of these changes were common among all the children and are summarized hereafter.

**Figure 5:**
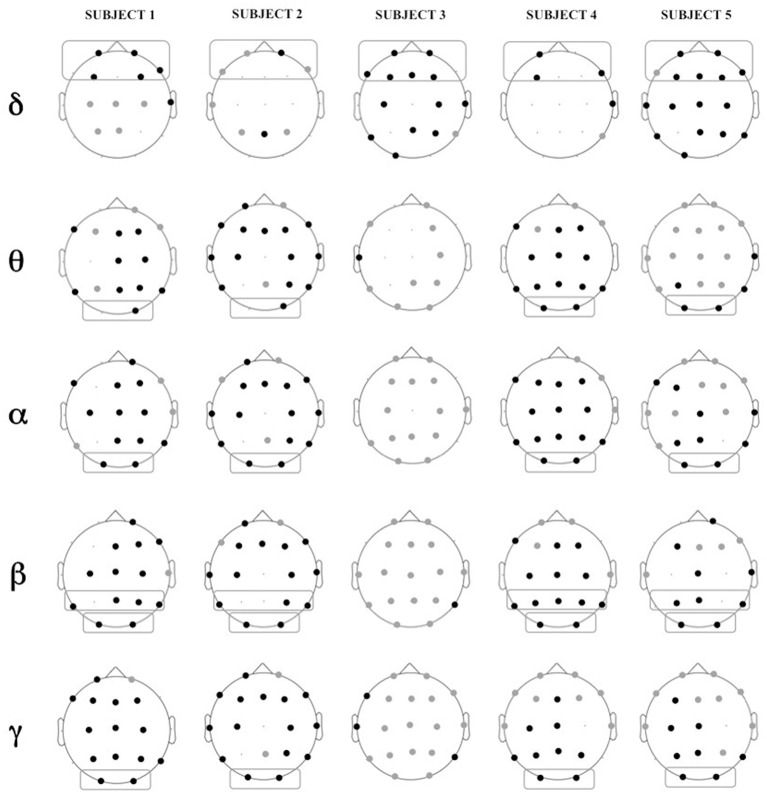
**Location of electrodes with significant differences in relative power in Engagement vs. Disengagement phase for each subject in each frequency band: black dots represent location of increase, gray dots location of decrease**. Boxes represent brain regions with significant increase in relative power at a group level analysis.

Figure 5 shows the location of the electrodes with statistically significant differences in PSD between Engagement and Disengagement phases.

Almost all the children displayed a significant relative power increase in most of the brain regions during the Engagement (Figure 5). At a group level analysis all the children showed significant power increase in frontal regions in delta band (*p* = 0.04). All but one child (Subject 3) also exhibited power increase in parietal regions in beta band (*p* = 0.04) and in occipital areas in theta, alpha, beta and gamma bands (*p* = 0.04). In Subject 3 there was not a significantly change in the PSD between Engagement and Disengagement in these frequency bands.

In the Engagement a transition from leftward to rightward asymmetry could be observed for almost all the frequency bands over the parietal areas. In particular this shift was significant in alpha and beta bands (*p* = 0.04). There was also a significant transition from rightward to leftward asymmetry in temporal areas in gamma band (*p* = 0.04).

**Figure 6:**
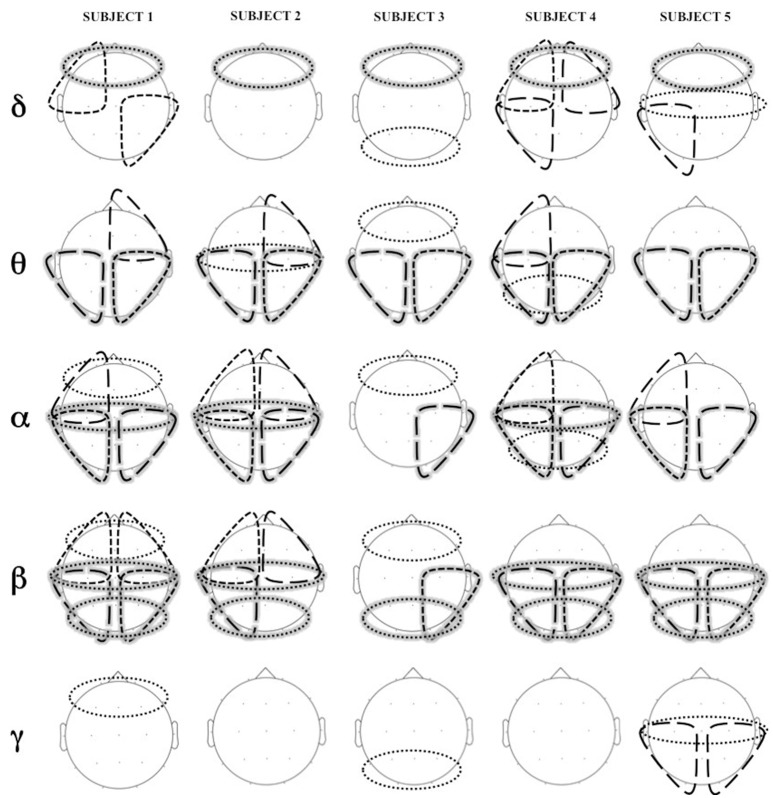
**Areas of significant increase in coherence in Engagement vs. Disengagement phase for each subject in each frequency band**. Long dashed lines represent transversal coherence while short dashed lines represent short coherence. Shaded areas represent regions of significant increase at a group level analysis.

Figure 6 shows statistically significant difference in coherence (coherence Engagement–coherence Disengagement) at the different cortical regions, in the different frequency bands.

A widespread increase of coherence was observed in almost all subjects during the Engagement in particular in theta, alpha and beta bands (Figure 6). The group level analysis revealed a significant increased in intrahemispheric coherence in posterior right and left areas in theta, alpha and beta bands (*p* = 0.04). Interhemispheric coherence increased at a central level in alpha and beta bands (*p* = 0.04) and at posterior level in beta band (*p* = 0.04). Although not significant at a group level analysis, it is possible to observe an increase of coherence in anterior regions in alpha band in almost all the subjects.

### ECG results

**Figure 7:**
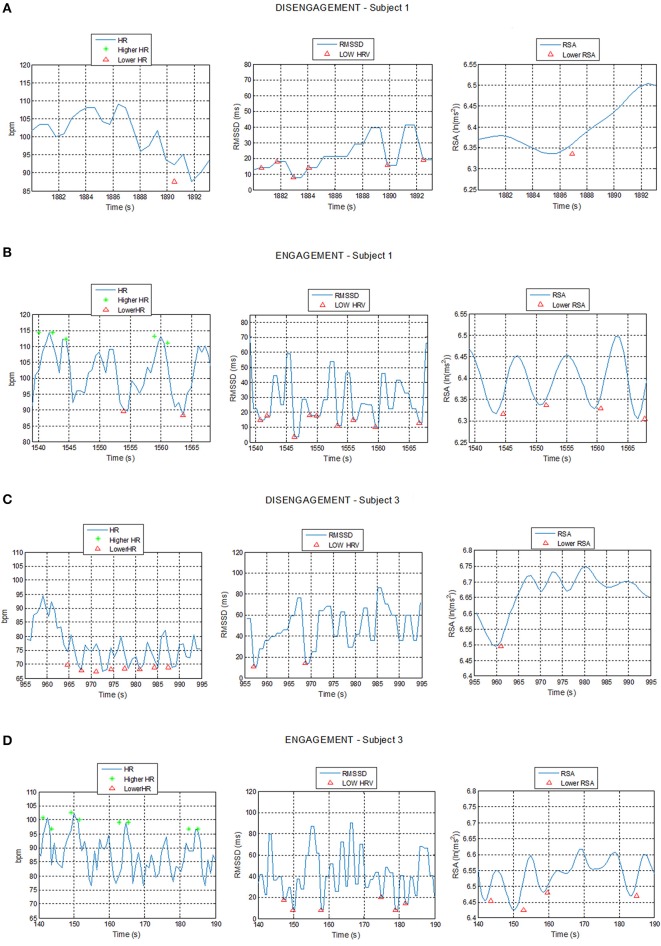
**Plot of Heart Rate (HR), Root Mean Square of the Successive Differences (RMSSD), and Respiratory Sinus Arrhythmia (RSA) trends during Disengagement (A,C) and Engagement (B,D) for Subject 1 and 3 with detection of physiological events: star markers represent HR value exceeding the high HR threshold while triangular markers represent HR, RMSSD, or RSA values undergoing the low HR, low RMSSD, and low RSA thresholds respectively**.

The system proved that the cardiac activity is strongly influenced by social engagement states and the ECG patterns turned out to be subjected to similar changes in the five subjects in response to changed psychological conditions. Figure 7 shows example of plots of the HR, RMSSD, and RSA trends over time during acquisition intervals for Subjects 1 and 3, corresponding to the Engagement and Disengagement phases. Markers locate physiological events according to the thresholds previously described. The ECG analysis showed a clear correlation between the detection of physiological events and the child's degree of involvement in the therapy. The plots reveal a greater presence of physiological events of “higher HR” and “lower HRV” and “lower RSA” in the Engagement as compared with the Disengagement. It was also evident that subjects displayed a remarkable RSA suppression compared with basal values in the Engagement. Furthermore, “lower HR” values turned out to be more frequent during the Disengagement.

Table 2 shows that children displayed an increased mean HR and a slightly significant greater percent number of “higher HR” physiological events during the Engagement compared to the Disengagement, excluding Subject 5 (*p* = 0.07). The mean RMSSD and mean RSA generally decreased during Engagement: therefore, the percent number of “lower HRV” and “lower RSA” events was significantly increased (*p* = 0.04 and *p* = 0.005 respectively).

**Table 2:** **Mean, Standard Deviation (SD), and percent number of physiological events for HR (bps), RMSSD (ms), and RSA (ln(ms^2^)) values during Engagement and Disengagement**.

**Subjects**	**Feature**	**Disengagement**		**Engagement**	
		**Mean ± SD**	**%Physiological events**	**Mean ± SD**	**%Physiological events**
Subject 1	HR	104.33 ± 3.21	15.8	104.68 ± 3.11	16.80
	RMSSD	36.22 ± 5.65	45.16	28.78 ± 5.53	47.64
	RSA	6.36 ± 0.05	3.22	6.36 ± 0.03	6.65
Subject 2	HR	97.64 ± 9.61	9.09	100.03 ± 9.29	16.63
	RMSSD	38.94 ± 14.12	42.13	44.69 ± 8.24	44.24
	RSA	6.44 ± 0.10	2.72	6.35 ± 0.25	4.47
Subject 3	HR	82.43 ± 2.93	3.43	85.26 ± 5.45	6.24
	RMSSD	48.41 ± 13.40	9.79	42.24 ± 8.79	13.4
	RSA	6.59 ± 0.03	7.90	6.57 ± 0.05	9.50
Subject 4	HR	92.58 ± 1.20	10.15	80.33 ± 0.41	15.20
	RMSSD	46.17 ± 2.35	10.35	30.60 ± 4.21	13.54
	RSA	6.45 ± 0.32	1.05	6.62 ± 0.26	5.04
Subject 5	HR	108.23 ± 0.23	66.65	95.39 ± 1.31	13.52
	RMSSD	17.15 ± 4.58	26.47	44.25 ± 3.53	66.54
	RSA	6.32 ± 0.12	1.01	6.47 ± 0.11	4.70

*Physiological events: higher HR, lower RMSSD, and lower RSA*.

## Discussion

The aim of this study was to develop and test innovative technologies to overcome some limitations of the present applications of physiological assessment in ASD (Bölte et al., 2016), including the artificial and constrained situations in which data are acquired and the need of a special compliance from the subjects, which prevented an ecological assessment so far.

This preliminary work represents an initial step to study the social cognition from an interactor point of view, based on the assumption that there is something fundamentally different when we are actively engaged with others in real-time social interaction as compared to when we merely observe them (Pfeiffer et al., 2012). The main contribution of this approach is that, if validated in a larger sample of subjects, would allow for more naturalistic paradigms that allow real world stimuli to be incorporated into EEG/psychophysiological studies.

In this pilot study, we have presented a paradigm for the acquisition of neurophysiological and physiological signals in a semi-naturalistic setting where children can interact with the examiner and play with different objects. Particular efforts have been provided to customize the ECG hardware, to integrate the videos, the ECG and the EEG units and to realize a user-friendly toolbox for data analysis.

The wireless EEG cap and the chest strap containing the ECG demonstrated how unobtrusive tools can be suitable for young children and can be used with semi-naturalistic paradigms. This study confirmed our previous results about the feasibility of the application of wearable sensors and wireless technologies in young subjects with neuropsychiatric disorders (Billeci et al., 2015). The interaction with the examiner rather than with a screen like in most EEG paradigms, is another very important feature of this study, and allows recreating a more real situation with social interactions and cues. In this paradigm, we can assume that the signals acquired from the brain and the autonomic system are much more similar to what is generated while the child interacts in common life situations. This setting, relatively simple to be implemented, can be considered as one step toward a more behaviorally-driven analysis of neurophysiological activity.

The acquisition of physiological signals during treatment could provide important cues about the response of the child, which is commonly just observed from a behavioral point of view by clinicians. This can be extremely important to objectivize the effect of the treatment and to implement more effective therapies.

In particular, literature shows the importance of the QEEG technique for assessing brain connectivity and for the development of an individualized treatment program (Billeci et al., 2013). Previous QEEG studies showed how autistic children have differences in power spectra, coherence, and symmetry measures with respect to controls (Cantor and Chabot, 2009). This is true both when signals are acquired in a resting condition, with open or closed eyes, and when specific tasks are performed (Cantor and Chabot, 2009). Furthermore, it has been observed that it is possible to link a specific pattern of brain activation, characterized by specific features, to certain specific behaviors.

In this study, we showed that with this approach is possible to measure changes in the EEG pattern during treatment elicited by interaction of the child with the therapist. An increase in relative power in Engagement compared with Disengagement emerged in the group analysis. The increase in frontal delta during cognitive tasks has been previously linked to perceptual switching of objects (Okada et al., 2009), an ability which is elicited during the imitation tasks with cubes during the imitation protocol. Occipital activity is mainly due to visual stimuli during the imitation task linked to tactile components (gamma and beta activity) (Bauer et al., 2009) and auditory component (alpha and theta) (Gladwin and de Jong, 2005). The activation of beta band within parietal regions has been linked to planning and execution of movement, although its functional role is still matter of debate (Zaepffel et al., 2013).

Interestingly, we also observed a general increase in coherence during Engagement. EEG coherence, being the covariance of spectral activity at two electrode sites, is a measure for the synchrony of neuronal activity and thus can be used as an indicator of effective cortical connectivity. Coherence mainly increased in theta, alpha and beta bands both intra- and inter-hemispheres. The increased coherence in these bands may reveal the increase of attention control during the participation in the cognitive task (Sacchet et al., 2015). Interestingly the level of coherence in frontal-parietal region in alpha band has been linked to different performances in an imitation task (Van der Helden et al., 2010). Moreover, several studies have shown a positive correlation between coherence in theta, alpha and beta and task associated with motor processing and execution (von Stein et al., 1999; Sauseng et al., 2005; Wheaton et al., 2005; Rilk et al., 2011).

A modification in asymmetry during Engagement was also observed. Previous studies have shown altered asymmetry in ASD brain both structurally and functionally. In particular, left-side prevalence in alpha and beta bands was found compared with controls both in visual and non-visual areas at rest. Right asymmetry in alpha and beta bands has been previously associated with discrimination of speech prosody (Kujala et al., 2005), processing gaze direction in face perception (Senju et al., 2005), and sustained visual attention (Stroganova et al., 2007). On the contrary, left temporal asymmetry in gamma band has been associated with language processing (Kojima et al., 2013) and an atypical asymmetry (rightward) of gamma band has been observed in ASD at rest (Maxwell et al., 2015). It seems from this study that the pattern of asymmetry shifts to the typical pattern during the Engagement phase.

It is worth noting that only two of the five subjects exhibit mu desynchronization during the imitation tasks, which could suggest a deficit in the mirror neuron system at least in some of the subjects enrolled in the study (MNS, Oberman et al., 2005). Given that all the children were able to perform the task is it possible that increase in alpha and beta coherence rather than mu desynchronization is a more reliable predictor of good performance in vasomotor tasks as suggested by Rilk et al. (2011).

ECG analysis confirmed previous findings and demonstrated that the system developed is able to detect ASD children's level of engagement (Park et al., 2013).

Some studies have shown that mental effort causes an increase in physiological arousal as measured for example by HR (Lundberg and Frankenhaeuser, 1980; Peters et al., 1998). Thus, subjective perception of mental effort may reflect changes in arousal during performance of attentive tasks. Precedent studies demonstrated how decreased RMSSD (Nagendra et al., 2015) and RSA (Overbeek et al., 2014) represented meaningful indicators for a positive response to attention demanding stimuli and is positively associated with cognitive function, including better processing speed, working memory, learning, and receptive language skills.

The ECG features could represent a valid marker to evaluate the engagement and the social interaction of the child in real time (Moore et al., 2009).

Importantly, besides showing some common pattern of modification in physiological parameters in the enrolled subjects, the implemented protocols allowed to evidence some inter-individual differences fostering the application of this platform for a personalization of the therapeutic protocol. In particular, as regards EEG it is clearly evident that Subject 3 did not showed significant differences between Engagement and Disengagement in many cortical areas. The clinical profile of this subject shows that he has an important concentration deficit and this can explain why, despite from the behavioral observation he seemed to be engaged in the tasks proposed by the therapist as the other subjects, his brain activated differently. In addition the desynchronization of the MNS is not observed in all the subjects. If the possibility of using the proposed system for characterizing the physiological profile of specific endophenotype of ASD would be confirmed in a larger sample, it would be very useful to guide the therapist, representing a step forward in the implementation of individual therapeutic programs in ASD.

Some important issues emerged from this study that suggest future developments. First of all, the need of a standardized, objective and clear distinction of the different behaviors of each task promotes rigorous segmentation of the signals. In the future annotation of the video could be done not only with manual markers but also with automatic or semi-automatic action recognition from the video recording.

Moreover, real-time feature extraction algorithms could be implemented, which provide the therapist a feedback about the status of the child. In this way the therapist could decide to potentiate some tasks of the therapy that cause a particular engagement of the child or, on the contrary, change the therapy if the child appears to be disengaged, stressed or not interested by the task proposed.

Overall, the system presented in this study was proven to be suitable for a similar clinical scenario. The children highly accepted the monitoring platform, as demonstrated by the low dropout rate in the study and by the fact that the children included in the study did not express any discomfort/annoyance at the system. Thus, the high grade of acceptability of the monitoring platform is promising for further development and implementation of the tool. Furthermore, the smart sensorized system allowed a reliable psychophysiological characterization of the children enrolled. Such evidence fosters the applicability of the system proposed in a clinical setting, where it could be used as a smart monitoring tool to support the clinicians during the treatment. In the future the proposed system could supply useful feedback to the therapist in the treatment of ASD and even in other neurodevelopmental disabilities.

Further data collections will be needed to confirm these preliminary results. At first, larger studies would be useful to generalize the findings of this feasibility pilot study and to obtain a clearer picture of the parameters examined in ASD children. Then, when confirmed on larger cohorts, the psychophysiological characterization of the children with ASD related to different behaviors will possibly allow to personalize the treatment and to longitudinally verify the treatment effect through objective measures of brain and autonomic function of children with ASD. In conclusion, this kind of evaluation holds promise for future developments in instrumental evaluations of ASD.

### Limitations

Some limitations need to be considered when interpreting the results. A first limitation is the small sample size. The study was designed as a feasibility study, thus only a small number of patients was recruited to test the applicability of the technology and the methodology.

The findings of the study need to be replicated with larger sample to prove the efficacy of the approach and the transferability in a clinical scenario. Larger sample will also allow for the evaluation of how different could be the physiological response of subgroups of children with ASD.

Another limitation is the absence of a control group, which prevents to discuss the nature of brain and autonomic response specific to ASD. In the future the comparison with a control group of age-matched children with typically development will allow to evaluate not only how different is the pattern between Engagement and Disengagement phases in ASD but also how this patterns are different from a typical pattern of response.

Finally another possible limitation is that we did not control for effects of physical movement. However, in our protocol the children were seated in a chair throughout the interaction and thus relatively restrained in their physical activity. As regards ECG, Porges et al. (2007) found that low intensity motor movements did not influence RSA or HRV in school-age children, so that movement were unlikely to have had a major impact on our results. As regards EEG we have removed muscular artifacts however visual inspection to is subjected to human error. In the future surface electromyography sensors could be used to control for children' muscular artifacts.

## Author contributions

LB developed EEG algorithms and analyzed data, participated in the development of the study design, contributed in the discussion and interpretation of the results, in writing and approving the paper. AT participated in the acquisition analysis of data, contributed in the discussion and interpretation of the result, in writing and approving the paper. GT developed the ECG algorithms and contributed in writing and approving the paper. AN performed therapies and coding of videos, participated in the development of the study design, and contributed in writing and approving the paper. SP analyzed ECG data and contributed in writing and approving the paper. DC developed the ECG communication protocol and approved the paper. GB developed the ECG software data and approved the paper. FC participated in the development of the study design, developed the video toolbox and approved the paper. SA participated in the development of technological platform and approved the paper. SC recruited the subjects and contributed in coding videos and in the discussion and interpretation of the result, in writing and approving the paper. GP participated in the development of the study design, was responsible of the technological protocol, and approved the paper. FM participated in the development of the study design, was responsible of recruitment and diagnosis of children and contributed in the discussion and approving the paper.

## Funding

The research leading to this work has received funding from the European Union's Seventh Framework Programme (FP7-ICT-288241) Project MICHELANGELO: Patient-centric model for remote management, treatment, and rehabilitation of autistic children. SC was supported by the Ministry of Health, Italy and by Tuscany Region with the grant “GR-2010-2317873,” and by Bando FAS Salute Sviluppo Toscana—ARIANNA Project.

### Conflict of interest statement

The authors declare that the research was conducted in the absence of any commercial or financial relationships that could be construed as a potential conflict of interest.

## Appendix

### Michelangelo study group

Silvio Bonfiglio (FIMI, Italy) Cristiano Paggetti (I+, Italy), Valentina Bono, Koushik Maharatna (University of Southampton, UK), Maryrose Francisa, Angele Giuliano (Across Limits, Malta), Mark Donnelly, Leo Galway (University of Ulster, UK), Fabio Apicella, Chiara Lucentini, Federico Sicca (Stella Maris Scientific Institute, Italy), Mohamed Chetouani (Université Pierre et Marie Curie, France), David Cohen, Jean Xavier (Groupe Hospitalier Pitié-Salpêtrière, France).
