# Supplementary material for: An Integrated Approach for the Monitoring of Brain and Autonomic Response of Children with Autism Spectrum Disorders during Treatment by Wearable Technologies
Source: Front Neurosci. 2016 Jun 21;10:276. doi: 10.3389/fnins.2016.00276 (PMC4914552; doi:10.3389/fnins.2016.00276)
Supplement: Supplementary file 1 [file DataSheet1.docx]

**Supplementary material**

**EEG features**

- The Brain Symmetry Index (BSI) is calculated as:

$$BSI=(LH-RH)/(LH+RH)$$

where LH and RH are the squared fast Fourier transform (FFT) coefficients of the signals from the left and right channels. BSI closed to 0 indicates perfect symmetry while values closed to 1 or -1 indicate maximal asymmetry (left or right-shifted).

- Coherence is calculated for each pair of electrodes to the normalized cross-spectral density function and is a measure of phase locking. The coherence between electrode 1 and electrode 2 is given by the following expression:

$$C_{12}=\frac{\left|P_{12}(f)\right|^{2}}{P_{11}(f)P_{22}(f)}$$

where $P_{12}(f)$ is the cross-spectrum for the two electrodes, while $P_{11}(f)$ and $P_{22}(f)$ are the spectra related to electrode 1 and electrode 2, respectively. Also, coherence is calculated within each frequency band of the QEEG signal. Coherence data are presented as a matrix for each band in which electrodes are displayed on the x-axis and on the y-axis. Elements of the matrix represent color-coded coherence values between the electrodes. The diagonal represents each channel’s coherence with itself, which is always 1.

For the purpose of data reduction, four EEG lead-groupings were considered to compute coherence (Figure 1): Inter-hemispheric Coherence, Intra-hemispheric Long-Range, Transverse Intra-hemispheric, and Intra-hemispheric Short-Range. For the inter-hemispheric values of coherence posterior (InterP), central (InterC) and frontal (InterF) brain regions were considered. Transverse intra-hemispheric coherences were calculated in four regions: anterior right (IntraTransvAntR), anterior left (IntraTransvAntL), posterior right (IntraTransvPostR), and posterior left (IntraTransvPostL) region. Short-range intra-hemispheric coherences were also calculated in four regions: anterior right (IntraShortAntR), anterior left (IntraShortAntL), posterior right (IntraShortPostR), and posterior left (IntraShortPostL). For the inter-hemispheric values of coherence a sagittal factor was considered, including posterior, central and frontal brain regions. For the anterior region the EEG Fp1– Fp2, F3–F4, and F7–F8 leads were analyzed. The EEG leads C3–C4 and T3–T4 values of coherence were considered for the central. A posterior region was also considered including the coherence values for the EEG derivations P3–P4, T5–T6, and O1–O2.

For the transverse intra-hemispheric coherence, four regions were considered: an anterior right for the EEG leads Fp2–F8, Fp2–T4, F4–F8, F4–T4, and C4–F8; an anterior left region with coherence values for Fp1–F7, Fp1–T3, F3–T3, and C3–F7 leads; a posterior right region including coherence values for C4–T6, P4–T4, P4–T6, O2– T4, and O2–T6 leads; and finally a posterior left region including coherence values for C3–T5, P3–T3, P3–T5, O1– T3, and O1–T5 derivations.

For the short-range intra-hemispheric coherences four regions were also considered: an anterior right region including Fp2–F4, F4–C4, and F8–T4 leads; an anterior left including values for Fp1–F3, F3–C3, and F7–T3 derivations; a posterior right region including T3–T5, C3–P3, and P3–O1 leads; and finally a posterior right region with coherence values for T4–T6, C4–P4, and P4–O2 derivations.


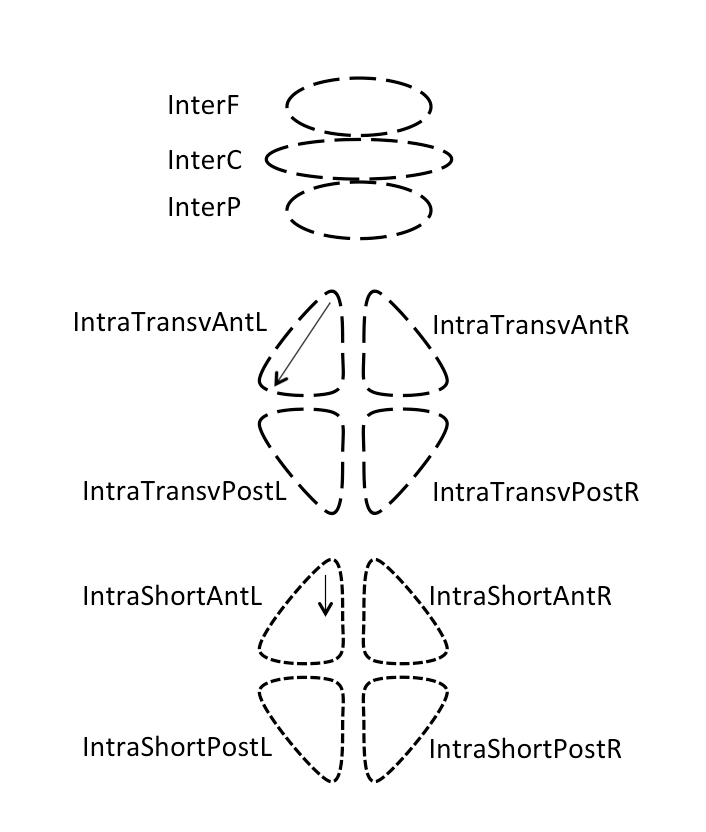


Supplementary Figure 4: Grouping of EEG leads for coherence analysis.

**ECG features**

- The Heart Rate (HR) is computed dividing 60 by each element of the Tachogram and a moving average is then applied in order to smooth out fluctuations and make the actual trend of the heart rate more visible in correspondence to the event markers.
- The Root Mean Square of Successive Difference (RMSSD) is described by the following equation:

$$RMSSD= \sqrt{\frac{1}{N-1}\sum_{i=1}^{N-1} \left( RR_{i+1}-RR_{i} \right)^{2}}$$

where N is the total number of all RR intervals (adjacent RR peaks) in the segment.

- The RSA component is extracted from the tachogram signal using the Empirical Model Decomposition (EMD), which is usually selected for nonlinear and nonstationary time series analysis. The EMD decomposes the tachogram into its components allowing the identification of the respiratory pattern associated with one of the oscillatory modes in the signal called Intrinsic Mode Function (IMF). The first IMF (IMF1) associated with the highest frequency contributing to HRV, was selected to extract the RSA feature.

The algorithm operates through six steps:

(1) Identification of all the extrema (maxima and minima) of the series X.

(2) Generation of the upper and lower envelope via cubic spline interpolation among all the maxima and minima, respectively.

(3) Point by point averaging of the two envelopes to compute a local mean series m.

(4) Subtraction of m from the data to obtain a IMF candidate h = X - m.

(5) Checking of the properties of h:

• if h is not a IMF (i.e. it does not satisfy the previously defined properties), replace X with h and repeat the procedure from Step 1;

• if h is a IMF, evaluate the residue r = X - h.

(6) Repeat the procedure from Steps 1 to 5 by sifting the residual signal. The sifting process ends when the residue r satisfies a predefined stopping criterion.
